# Pharmaceutical Assessment of *Melia azedarach* Gum as a Binder and Disintegrant in Immediate-Release Tablets

**DOI:** 10.1155/2022/9810099

**Published:** 2022-04-01

**Authors:** Frederick William Akuffo Owusu, Mariam El Boakye-Gyasi, Raphael Johnson, Yaa Asantewaa Osei, Emmanuel Asante, Desmond Asamoah Bruce Otu, Julia Afrakoma Ansong, Philomena Entsie, Marcel Tunkumgnen Bayor

**Affiliations:** ^1^Department of Pharmaceutics, Faculty of Pharmacy and Pharmaceutical Sciences, Kwame Nkrumah University of Science and Technology, Kumasi, Ghana; ^2^Department of Herbal Medicine, Faculty of Pharmacy and Pharmaceutical Sciences, Kwame Nkrumah University of Science and Technology, Kumasi, Ghana

## Abstract

Excipients are components other than active ingredients that are added to pharmaceutical formulations. Naturally sourced excipients are gradually gaining preeminence over synthetically sourced excipients due to local availability and continuous supply. This study aimed to investigate the binding and disintegrating characteristics of gum extracted from the bark of *Melia azedarach* tree. The bark of *Melia azedarach* was harvested from Kwahu Asasraka in Ghana. The gum was extracted with ethanol (96%), and the percentage yield, phytochemical constituents, and flow characteristics were assessed. As a disintegrant, the gum was utilized to formulate granules at varying concentrations of 5% w/w and 10% w/w using starch as the standard. The gum was also utilized to prepare granules at varying concentrations of 10% w/v and 20% w/v as a binder, with tragacanth gum serving as the reference. Eight batches of tablets were produced from the granules. The formulated tablets from each batch were then subjected to quality control testing, which included uniformity of weight, friability, disintegration, hardness, drug content, and dissolution tests, respectively. Tannins, saponins, alkaloids, and glycosides were identified in the *Melia azedarach* gum. The gum had a percentage yield of 67.75% and also exhibited good flow properties. All tablets passed the uniformity of weight, friability, disintegration, hardness, dissolution, and drug content tests, respectively. According to the findings of the study, *Melia azedarach* gum can be utilized as an excipient in place of tragacanth and starch as a binder and disintegrant, respectively, in immediate-release tablets.

## 1. Introduction

The oral route is the most common route of medication administration, and tablets in different forms are the most common oral dosage form [1]. Tablets are classified into various types, including modified-release and immediate-release tablets with the latter being the most common [1, 2]. When taken into the body, immediate-release tablets are meant to disintegrate and release the drug with no specific rate regulating characteristics such as special coatings. These tablets disintegrate quickly and dissolve, allowing the medications to be released [2].

When placed in a fluid environment, disintegrants help in the breakdown of the compacted mass of the immediate-release tablets [3]. Disintegrants among other mechanisms predominantly enhance medication release by increasing water wicking into the plug and promoting the deaggregation of the plug particles [3, 4]. They can be derived from natural or synthetic sources [5]. Binders are chemicals that are added to powdered mixes to guarantee that formulated granules and tablets have the necessary mechanical strength when produced [1, 6]. Binders are also harnessed from natural and synthetic sources. Binders and disintegrants obtained from natural sources are being sought after in recent times in comparison to their synthetic counterparts due to ease of availability, low cost, eco-friendliness, ease of degradability, and compatibility due to their natural nature [2, 5–7]. Even though gums and mucilage are composed mostly of polysaccharides, proteins, and urbanites, mucilage is produced during normal plant development by mucilage secreting glands, while gums are thought to be pathological compounds produced as a result of mechanical injury to certain plants or undesirable climatic conditions, such as drought [6–8]. Gums and mucilage obtained from several trees and shrubs in Ghana have been investigated for their potential as pharmaceutical excipients due to the high cost involved in the importation of natural gums such as tragacanth and acacia [9]. However, *Melia azedarach* is one of the commonest trees in Ghana whose gum has not been fully explored as a potential pharmaceutical excipient in solid dosage forms. Folklorically, different parts of the tree are used in treating several disease conditions such as worm infestations, amenorrhea, and as a diuretic agent [10]. In this research, the binding and disintegrating properties of the gum derived from the *Melia azedarach* tree were investigated as a means of obtaining alternative sources of pharmaceutical excipients for immediate-release tablets. Currently, to the best of our knowledge, this is the first study reporting on the potential of *Melia azedarach* gum as a binder and a disintegrant in immediate-release tablets.

## 2. Materials and Methods

### 2.1. Materials

*Melia azedarach* gum was obtained from Kwahu Asasraka in the Eastern Region of Ghana and authenticated at the Department of Pharmacognosy, KNUST. Other materials used include paracetamol powder (Shanghai Dumi Biotechnology), tragacanth gum powder (Xiʼan Rongsheng Biotechnology), starch (Jilin COFCO Bio-Chem), and lactose (Xiʼan Rongsheng Biotechnology). All other reagents were of analytical grade and were used as received.

### 2.2. Methodology

#### 2.2.1. Extraction of Gum from the Bark of *Melia azedarach*

In a conical flask, 100 g of powdered *Melia azedarach* crude gum was dissolved in 1500 mL of distilled water and left to stand for 24 hours at room temperature. The gum mucilage was filtered through a calico cloth, which was gripped and pressed forcefully to remove any insoluble material. To guarantee that all debris was eliminated, the filtered mucilage was refiltered. The filtrate was then washed with diethyl ether after being precipitated with three times the amount of 96% ethanol. The purified gum was dried for 24 hours in a hot air oven at 40°C. The dry gum was milled and sieved with a sieve with an aperture size of 180 µm. The powdered gum was sealed in an airtight container and kept in a cool, dry place [11].

#### 2.2.2. Determination of Percentage Yield of *Melia azedarach* Gum

An analytical balance was used to quantify the weight of powdered *Melia azedarach* gum (W1) and dried purified gum (W2). The percentage yield was then calculated using the following formula:

(1) $$\begin{array}{c} \%\text{ yield}=\frac{\text{weight before purification}}{\text{weight after purification}}x\;100. \end{array}$$

#### 2.2.3. Phytochemical and Physicochemical Analysis of Powdered *Melia azedarach* Gum

Phytochemical tests (for alkaloids, tannins, and glycosides) and physicochemical (elemental contents and swelling index) tests were carried out on the extracted gum using previously established methods [12–15].

#### 2.2.4. Granules Formulation

The wet method of granulation (using water as the granulating liquid) and the technique of doubling the bulk were employed in preparing the paracetamol granules using *Melia azedarach* gum as a binder and a disintegrant [15]. The gum was used as a disintegrant at concentrations of 5% and 10% w/w (F1 and F2) in formulating the paracetamol with starch used as the standard (F3 and F4). The gum was used as a binder at concentrations of 10%w/v and 20%w/v (F7 and F8) with tragacanth used as the standard (F5 and F6). The formula for preparing the granules is summarized in Table 1. The quantities were scaled up to prepare granules for a hundred (100) tablets. The granules were compressed into tablets using a single punch tableting machine Saimach (11/37).

#### 2.2.5. Flow Properties and Compressibility Index

In assessing the flow properties, the fixed height method was used for the angle of repose determination, while Carr's index and Hausner's ratio were determined with the aid of bulk density (Db) and tapped density (Dt) parameters [16].

#### 2.2.6. Evaluation of Tablet Properties

*Physicomechanical Properties*. The physicomechanical parameters such as uniformity of weight, dimensions, hardness, friability, and disintegration were analyzed according to the methods stated by [15].

*Calibration Curve for Paracetamol*. A paracetamol solution (0.15% w/v) was prepared with phosphate buffer (pH 5.8) to a volume of 200 mL. Serial dilutions with concentrations of 0.0004, 0.0005, 0.0006, 0.0007, and 0.0008% w/v were prepared. The absorbances were spectrophotometrically determined at a maximum wavelength of 245 nm. The blank used was phosphate buffer of pH 5.8. The relationship between the concentration and the absorbance was plotted to obtain a calibration curve (y = 491.15x – 5E-05, R^2^ = 0.9996) [17].

*Assay*. As described by the BP, an equivalent weight of a tablet from a batch corresponding to a dose in one tablet was weighed from ten crushed tablets and dissolved in 75 mL of phosphate buffer (pH 5.8), which was then filled up to 100 mL and filtered. With the phosphate buffer, the filtrate was filled to a volume of 100 mL. Serial dilutions were then prepared, and the absorbance and drug content were evaluated using the precomputed maximum wavelength and the calibration curve [18]. Each batch was held to the same standards.

*Dissolution Studies*. The dissolution test was conducted using the USP dissolution apparatus II at 50 rpm. Six (6) formulated tablets each from all batches were placed in a vessel containing 900 mL of phosphate buffer (pH 5.8) for *in vitro* dissolution experiments and controlled at 37 ± 0.5°C. At 5, 15, 30, 45, and 60 minutes, a volume of medium (10 mL) was drawn midway between the medium and the upper portion of the spinning paddle blade, not less than one centimeter from the vessel wall, and filtered using Whatman filter paper. To replace the portions removed, a volume of 10 mL of fresh medium was added. The absorbance of the filtrates at 245 nm was measured using a UV-visible spectrophotometer. The concentrations of paracetamol extracted from the tablets were calculated using the equation derived from the calibration curve at 0, 5, 15, 30, 45, and 60 minutes [15, 19].

*Data Analysis*. The mean and standard deviation are used for the data representation. GraphPad Prism version 6.00 for Windows (GraphPad Software, San Diego, California, USA) was used to analyze the data. At a 95% confidence interval, *p* < 0.05 was judged significantly different.

## 3. Results and Discussion

### 3.1. Percentage Yield and Macroscopic Properties of *Melia azedarach* Gum

The extraction resulted in a percentage yield of 67.8 ± 0.11. This value was higher than the yield of gum obtained from *Albizia* (39.38%) and *Khaya* (67.50%) trees as reported by Bonsu and others (2016) [20]. The yield achieved may be influenced by the geographical location, season, extraction process, and solvent employed. The gum of *Melia azedarach* was yellowish-brown in colour, odourless, and smooth in texture.

Tannins, saponins, alkaloids, and glycosides were all present in the *Melia azedarach* gum (Table 2). Venkatachalam et al. (2020) [21] have reported that the extractive processes used in obtaining purified gums from their crude forms can result in the loss of some phytochemical constituents. Tannins have been found to possess antioxidant properties [22]. This means that when the gum is incorporated into the pharmaceutical formulation, it will be able to prevent the gum from reacting with oxygen to change the pharmaceutical product. Saponins have antimicrobial activity which means that the gum can potentially inhibit microbes when it is used in pharmaceutical preparations [23]. Glycosides have both antioxidant and antimicrobial activities, and hence when incorporated, the gum will have the ability to inhibit microbes and also help prevent oxidation reaction occurring between the gum and oxygen to cause a change in the product [24]. These further enhance the suitability of *Melia azedarach* gum as a pharmaceutical excipient.

### 3.2. Metal Ions Analysis and Swelling Index of *Melia azedarach* Gum

The swelling capacity of excipients incorporated as binders and disintegrants in immediate-release tablets is important since the dosage form must absorb water, expand in size, and disintegrate in order to release the medication for dissolution and absorption. The *Melia azedarach* gum exhibited excellent swelling properties of 81.23 ± 0.086 which was higher than gum from other sources [20]. This is an indication of its potential as a binder and disintegrant in immediate-release tablets.

Elemental analysis of the gum showed the presence of minerals including nitrogen, phosphorus, potassium, calcium, magnesium, and sodium. Micronutrients such as copper, iron, zinc, and manganese were also detected (Table 3). All elements identified were within specifications which indicates the suitability of *Melia azedarach* gum as a pharmaceutical excipient. Furthermore, *Melia azedarach* gum was observed to have higher levels of micronutrients and minerals as compared to other gums from natural sources (*Khaya and Albizia*) reported by [20].

### 3.3. Physicomechanical Properties of Formulated Paracetamol Tablets

The physical and mechanical characteristics of the various batches of paracetamol tablets produced are summarized inTables 4 and 5.  Table 4 shows that the granules produced prior to compression have good flow characteristics with the exception of F7 which had poor flow [15]. The granules with high flow characteristics contain lower particle sizes and a combination of coarse and fine aggregates, allowing for improved flow. This will improve tablet compression by reducing the amount of gravitational force necessary for the granules to move from the hopper to the die. The free flow of the granules is also influenced by the cohesiveness of the particles in the granules [25].

A batch of tablets with an average weight of more than 250 mg conforms to the British Pharmacopoeia standard if no more than 2 tablets are outside the percentage limit of ±5% and no tablet differs by more than twice (±10%) the permissible limit.  Table 5 shows that all of the batches passed the uniformity of weight test; none of the batches had two tablets that were outside the% limit, and none of the batches had a tablet that was more than twice the percentage limit. This implies that the active pharmaceutical ingredients (APIs) and other excipients are evenly dispersed throughout the tablet [26].

The friability index of uncoated immediate-release tablets should not be more than 1% according to the USP. All the batches passed the friability test (using ten tablets). None of the batches had a percentage higher than 1% (Table 5). This means that the tablets produced with *Melia azedarach* gum have good mechanical strength to overcome abrasion during handling, packing, and transportation [27].

**Figure 1:**
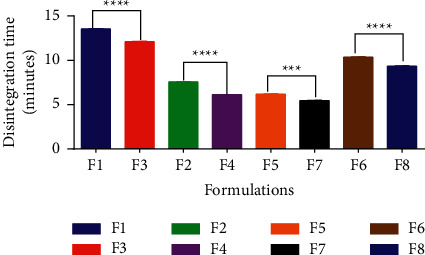
Statistical analysis of the disintegration time of formulated tablets using Student's two-tailed test. ^∗∗∗∗^*p* ≤ 0.0001 and ^*∗∗∗*^*p* ≤ 0.001. The values are mean ± SD, *n* = 3.

When placed in physiological solution, uncoated immediate-release tablets should disintegrate within 15 minutes, according to the USP specifications. All of the batches passed the disintegration test (Table 5). All batches of tablets containing *Melia azedarach* gum as a disintegrant had a disintegration time of less than 15 minutes and less than that of the standard binder and disintegrant with significant differences (P˂0.05) (Figure 1), indicating that *Melia azedarach* gum is a good disintegrant and a good binder which will be able to break up in the gastrointestinal tract (GI) to release the active pharmaceutical ingredient. This may be because the gum has a high swelling index [28]. According to the USP, the crushing strength of an uncoated immediate-release tablet should be within the range of 4–8 kg. From the results obtained in this study (Table 5), all the batches passed the hardness test.

This indicates that *Melia azedarach* gum-based tablets can withstand fracture during handling, storage, and transit [19]. From the results obtained (Table 5), it was realized that increasing the concentration of *Melia azedarach* gum as a binder results in an increase in tablet hardness which is a common property of binders used in tablets.

**Figure 2:**
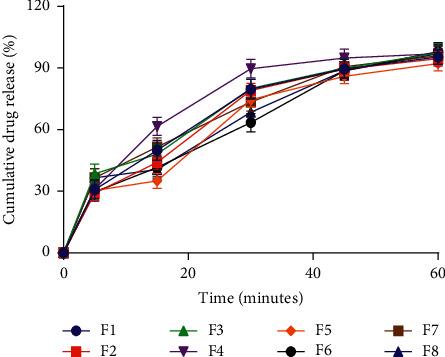
Drug release profile of formulated paracetamol tablets. The values are mean ± SD, *n* = 6.

All batches had drug content in the range of 97% to 102%, indicating that the tablet will be able to perform its therapeutic function [28]. According to the British Pharmacopoeia, the amount of medication dissolved after 45 minutes should not be less than 70%. All formulated batches passed the dissolution test; each batch had more than 70 percent of the active components dissolving after 45 minutes (Figure 2). The dissolution rate is directly related to the drug's bioavailability and is a good index for comparing the bioavailability of two tablets of the same drug. The absorption rate and the degree of the resulting therapy are influenced by the dissolution rate [29–31]. The dissolution rate influences the absorption rate and the degree of the resulting therapeutic effect of a drug, and it is affected by the binder form and concentration, hardness, surface area, diffusion distance, production process (wet granulation, dry granulation, or direct compression), and diluents [20]. Therefore, incorporation of *Melia azedarach* gum as a binder and disintegrant produces tablets with the desired dissolution profile for therapeutic activity to be achieved. Furthermore, tablets formulated from *Melia azedarach* gum released paracetamol in similar amounts to tablets made from starch and tragacanth at all concentrations tested (Table 6). As a result, *Melia azedarach* gum can be used as a substitute for tragacanth and starch in the manufacture of bioequivalent immediate-release tablets [30].

## 4. Conclusion

Tablets compressed with *Melia azedarach* gum as a binder (10%w/w and 20%w/v) and a disintegrant (5%w/w and 10%w/w) passed all quality control tests carried out on them and were comparable to a standard disintegrant (starch) and binder (tragacanth), respectively. Ultimately, gum obtained from *Melia azedarach* can be incorporated as a binder and disintegrant in immediate-release tablets with properties comparable to tragacanth and starch, respectively.

## Figures and Tables

**Table 1:** Master formula for preparation of granules.

Formulation code	Ingredients (mg/tablet)					
	Disintegrant	Disintegrant	Binder	Binder	Diluent	Lubricant and glidant
	*Melia* gum	Starch	Tragacanth	*Melia* gum	Lactose	Talc
F1	30.0	-	10.0	-	70.0	0.5
F2	60.0	-	10.0	-	40.0	0.5
F3	-	30.0	10.0	-	70.0	0.5
F4	-	60.0	10.0	-	40.0	0.5
F5	-	45.0	25.0	-	55.0	0.8
F6	-	45.0	50.0	-	55.0	0.8
F7	-	45.0	-	25.0	55.0	0.8
F8	-	45.0	-	50.0	55.0	0.8

Each batch contained paracetamol (500 mg) as the active pharmaceutical ingredient (API).

**Table 2:** Macroscopic properties and phytochemical constituents of the gum of *Melia azedarach*.

Macroscopic		Phytochemicals		
Characteristics	Observation	Phytochemical	Observation	Inference
Colour	Brownish	Tannins	White ppt formed	Present
Odour	Odourless	Saponins	Persistent froth	Present
Taste	Bland	Alkaloids	Brown ppt formed	Present
Texture	Smooth	Glycosides	Brick-red ppt formed	Present

Ppt : precipitate.

**Table 3:** Metal ions analysis of *Melia azedarach* gum.

Parameter (mg/kg)	*Melia azedarach* gum
N (mg/kg)	2,858.04 ± 46.67
P (mg/kg)	465.77 ± 64.46
K (mg/kg)	1,388.83 ± 41.71
Ca (mg/kg)	14,404.00 ± 73.92
Mg (mg/kg)	3,360.00 ± 54.32
Na (mg/kg)	480.19 ± 23.76
Fe (mg/kg)	65.28 ± 3.53
Cu (mg/kg)	23.77 ± 2.66
Zn (mg/kg)	36.37 ± 2.49
Mn (mg/kg)	48.25 ± 6.63

The values are mean ± SD, *n* = 3.

**Table 4:** Flow properties of formulated granules from *Melia* gum.

Formulation	Bulk density (g/mL)	Tapped density (g/mL)	Carr's index (%)	Hausner's ratio	Angle of repose (*θ*)
F1	0.471 ± 0.02	0.544 ± 0.01	13.419 ± 0.02	1.155 ± 0.02	23.199 ± 0.02
F2	0.466 ± 0.01	0.596 ± 0.02	21.812 ± 0.01	1.279 ± 0.02	25.821 ± 0.01
F3	0.414 ± 0.02	0.517 ± 0.03	19.923 ± 0.02	1.246 ± 0.01	26.565 ± 0.02
F4	0.409 ± 0.01	0.516 ± 0.02	20.736 ± 0.01	1.262 ± 0.01	26.565 ± 0.01
F5	0.394 ± 0.03	0.466 ± 0.01	15.451 ± 0.02	1.183 ± 0.02	26.565 ± 0.02
F6	0.405 ± 0.02	0.501 ± 0.01	19.162 ± 0.01	1.237 ± 0.01	27.759 ± 0.01
F7	0.413 ± 0.01	0.631 ± 0.02	35.548 ± 0.02	1.528 ± 0.02	33.690 ± 0.03
F8	0.420 ± 0.01	0.588 ± 0.01	28.751 ± 0.03	1.400 ± 0.02	27.759 ± 0.01

The values are mean ± SD, *n* = 3.

**Table 5:** Physicomechanical properties of the batches of paracetamol tablets produced.

Batch No.	Friability (%)	Assay (%) *n* = 3	Average weight (g) *n* = 20	Hardness (kg/f) *n* = 6	Disintegration time (min) *n* = 3
1	0.1	98.70 ± 0.01	0.639 ± 0.00	4.740 ± 0.08	13.55 ± 0.01
2	0.1	99.50 ± 0.09	0.639 ± 0.00	4.722 ± 0.03	7.55 ± 0.01
3	0.1	97.21 ± 0.05	0.637 ± 0.00	4.630 ± 0.03	12.12 ± 0.01
4	0.2	99.20 ± 0.09	0.639 ± 0.00	4.775 ± 0.06	6.12 ± 0.01
5	0.1	99.81 ± 0.08	0.629 ± 0.00	4.850 ± 0.06	6.20 ± 0.01
6	0.1	99.61 ± 0.07	0.639 ± 0.00	5.027 ± 0.08	10.36 ± 0.02
7	0.1	97.81 ± 0.06	0.630 ± 0.00	4.827 ± 0.05	5.46 ± 0.03
8	0.1	99.42 ± 0.04	0.630 ± 0.00	4.807 ± 0.06	9.34 ± 0.04

**Table 6:** Difference (*f1*) and similarity (*f2*) factors of formulated batches of *Melia azedarach* gum in comparison with standard binder (tragacanth) or disintegrant (starch).

Formulation	Difference factor (*f1)*	Similarity factor (*f2*)	Comment
F3	3.53	71.02	Similar
F4	11.20	51.20	Similar
F7	9.74	54.48	Similar
F8	2.60	71.06	Similar

## Data Availability

The data used to support the findings of this study are included within the article and are also available from the corresponding author upon request.
